# Strontium Phosphate Composite Designed to Red-Emission at Different Temperatures

**DOI:** 10.3390/ma13204468

**Published:** 2020-10-09

**Authors:** Katarzyna Szyszka, Adam Watras, Rafal J. Wiglusz

**Affiliations:** Institute of Low Temperature and Structure Research, Polish Academy of Sciences, Okolna 2, 50-422 Wroclaw, Poland; a.watras@intibs.pl

**Keywords:** luminescence, strontium hydroxyapatite, strontium phosphate, europium (III) ions, biomaterial, hydrothermal synthesis, Kröger–Vink-notation

## Abstract

Eu^3+^-doped Sr_10_(PO_4_)_6_(OH)_2_–Sr_3_(PO_4_)_2_ (SrHAp-TSP) composites were obtained via the microwave-stimulated hydrothermal method and post-heat-treated from 750 to 950 °C. Concentration of the Eu^3+^ ions was set to be 0.5, 1, 2, 3, 5 mol% in a ratio of the strontium ions molar content. The structural and morphological properties were investigated by X-ray powder diffraction (XRPD), scanning electron microscopy (SEM) and fourier transform infrared spectroscopy (FT-IR) techniques. The average particle size of the studied materials annealed at 750, 850 and 950 °C were counted about 100, 131 and 173 nm, respectively. The luminescence properties depending on the dopant ion concentration, heat-treatment temperature, excitation wavelength and temperature were investigated. In the emission spectra, a broad peak corresponding to the 4f^6^5d^1^ → 4f^7^ (^8^S_7/2_) emission of Eu^2+^ ions as well as narrow 4f-4f transitions typical for Eu^3+^ ions can be observed. The luminescence intensity of the 1 mol% Eu^3+^:Sr_10_(PO_4_)_6_(OH)_2_–Sr_3_(PO_4_)_2_ was measured depending on the ambient temperature in the range of 80–550 K. The CIE 1931 (International Commission on Illumination) chromaticity diagram was determined from emission spectra measured in 80, 300 and 550 K. The reduction mechanism of the Eu^3+^ to the Eu^2+^ was explained by the charge compensation mechanism based on the Kröger–Vink-notation. The decay times were measured and the Judd–Ofelt (J–O) theory was applied to analyze the observed structural and spectroscopic features.

## 1. Introduction

Apatite (Ap) is a large family of compounds commonly occurring in nature. Calcium hydroxyapatite (Ca_10_(PO_4_)6(OH)_2_ – CaHAp) is ideal representative of this family of minerals, which can be easily doped with different kinds of ions [1,2]. Apatite is applied in plenty industrial fields as catalysts, laser hosts, luminescent materials, gas sensors, ionic conductors and adsorbents [3,4]. Moreover, calcium phosphates are also used in implantology due to high similarity to the inorganic part of hard tissues in bones, high biocompatibility, osteoconductivity, nontoxicity, non-immunogenicity and bioactivity [5,6,7] Apatite doped with lanthanide ions are extensively investigated as bio-labels that can be used in in vitro and in vivo imaging [8,9,10].

Strontium is a trace element present in human bones and tooth impacting on the strength, healing, microarchitecture, and bone formation. Besides, strontium (II) ions were employed in osteoporosis therapy in the form of orally administrated strontium ranelate salt [11]. Moreover, strontium apatites seem to be good candidates as host lattices for luminescent dopants due to their excellent thermal and chemical stability as well as excellent luminescence properties. As an example, the Sr_5_(PO_4_)_3_Cl: Eu^2+^ material was used as the blue component in fluorescent lamp [12].

Designing of phosphor materials activated by lanthanide ions has enabled their applications in such technologies as solid-state lighting, flat panel displays, light-emitting diodes (LEDs), biological diagnostic, and biomedical areas due to their excellent properties [13]. Eu^3+^ ions are the most frequently used activator applied as red-emitting phosphor e.g., in commercially available phosphors Y_2_O_2_S: Eu^3+^ and Y_2_O_3_: Eu^3+^ [14,15]. Moreover, europium can occur in Eu^2+^ (f^7^) and Eu^3+^ (f^6^) oxidation states of suitable stability. Both show many different features as emitting centers. The Eu^2+^ ion is sensitive to oxidation under some conditions; however, it possesses good stability in the solid-state materials. It is of particular interest because it exhibits strong and tunable emission from blue to red [16]. The emission spectrum consists of the broad band related to the allowed 4f^6^5d^1^ → 4f^7^ (^8^S_7/2_) transition. Its luminescence properties strongly depend on its environment in the host lattice. The Eu^3+^ ion is also characterized by unique optical properties with a relatively strong red-orange emission. Its luminescence results from forbidden, intra-configurational f-f transitions roughly independent on crystal field influence. However, the intensity of particular transitions are highly sensitive to Eu^3+^ ions surrounding, so additionally, Eu^3+^ ions are very useful as a luminescence probe [17,18,19]. 

Luminescence materials co-doped with Eu^3+^ and Eu^2+^ is tough to obtain because the reduction of oxidation state in solid states requires a thermal treatment in reducing atmosphere (H_2_, H_2_/N_2_, CO). However, it is interesting that Eu^2+^-doped materials can be obtained in a nonreducing atmosphere at elevated temperatures in certain compounds due to reduction of Eu^3+^ ions to Eu^2+^ ions by the crystallographic site [20]. This abnormal reduction phenomenon was observed in crystal structures with BO_4_, SiO_4_, AlO_4,_ or PO_4_ tetrahedral groups.

In the present work, for the first time to the best of our knowledge [7,8,21,22,23,24], synthesis of strontium phosphate composite (Sr_10_(PO_4_)_6_(OH)_2_–Sr_3_(PO_4_)_2_) co-doped with Eu^3+^ and Eu^2+^ ions prepared in the air at high temperature and the structural, morphological as well as spectroscopic properties have been studied.

## 2. Materials and Methods

### 2.1. Synthesis of Composite

Strontium phosphate (Sr_10_(PO_4_)_6_(OH)_2_–Sr_3_(PO_4_)_2_) composite doped with Eu^3+^ ions was synthesised by microwave stimulated hydrothermal method and then post-treated at elevated temperature [7,8]. The Sr(NO_3_)_2_ (99.0% min Alfa Aesar, Karlsruhe, Germany), (NH_4_)_2_HPO_4_ (≥ 99.0% Fluka, Bucharest, Romania), Eu_2_O_3_ (99.99% Alfa Aesar, Karlsruhe, Germany) and NH_3_∙H_2_O (99% Avantor, Gliwice, Poland) for pH modification were used as substrates. The Eu^3+^ ions concentration was set on 0.5, 1, 2, 3 and 5 mol% in proportion of strontium ions molar content. Firstly, the stoichiometric Eu_2_O_3_ mass was digested in the HNO_3_ (ultrapure Avantor, Gliwice, Poland) to receive Eu(NO_3_)_3_·*x*H_2_O. Europium nitrate hydrate was re-crystallized three times in order to eliminate the HNO_3_ excess. Then, the stoichiometric Sr(NO_3_)_2_ mass was dissolved in water and the Eu(NO_3_)_3_·xH_2_O also was added in. Thereafter, 0.5353 g (4.05 mmol) of (NH_4_)_2_HPO_4_ was put to the previous mixture and the pH value was modified to 10 by means of ammonia. The suspension was transferred into Teflon vessel (Ertec, Wroclaw, Poland) and placed in the microwave hydrothermal reactor (MV 02-02, Ertec, Wroclaw, Poland). The mixture was heat treated at 280 °C for 90 min under 60 atm autogenous pressure. The obtained product was rinsed with de-ionized water (Hydrolab, Straszyn, Poland) several times and dried at 70 °C for 24 h. The thermal treatment from 750 to 950 °C for 3 h was the last step during materials preparation.

### 2.2. Characterisation

Powder diffraction patterns were received by using an X’Pert Pro diffractometer (PANalytical, Almelo, The Netherlands) equipped with Ni-filtered Cu Kα radiation (λ = 0.154 nm, V = 40 kV, I = 30 mA). The structural refinement was done by using a Maud program version 2.93 (University of Trento – Italy, Department of Industrial Engineering, Trento, Italy) [25,26] based on the apatite hexagonal and trigonal tristrontium diphosphate crystal structures with better approximation and indexing of the Crystallographic Information File (CIF). The quality of structural refinement was supervised by R-values (R_w_, R_wnb_, R_all_, R_nb_, and σ), which were followed to get a structural refinement with better quality and reliability.

A FEI Nova NanoSEM 230 scanning electron microscope (Hillsboro, OR, USA) equipped with energy dispersive spectrometer spectrometer (EDS; EDAX Genesis XM4; Hillsboro, OR, USA) and operating at an acceleration voltage in the range 3.0–15.0 kV and spot 4.0–4.5 was used to determine the surface morphology and the elements mapping. The EDS analysis was done to confirm the contents of elements in the obtained materials.

A Nicolet iS50 FT-IR (Thermo Scientific, Waltham, MA, USA) spectrometer equipped with an Automated Beamsplitter exchange system (iS50 ABX containing DLaTGS KBr detector) (Thermo Scientific, Waltham, MA, USA) and HeNe laser as an IR radiation source were used to measure IR spectra. Polycrystalline mid-IR spectra were collected in the 4000–400 cm^−1^ range in KBr pellets at the temperature of 295 K and spectral resolution of 4 cm^−1^.

The excitation spectra were recorded using an FLS980 Fluorescence Spectrometer (Edinburgh Instruments, Kirkton Campus, UK) equipped with 450 W Xenon lamp. The excitation of 300 mm focal length monochromator was in Czerny–Turner configuration. The excitation arm was supplied with holographic grating of 1800 lines/mm grating blazed at 250 nm. The excitation spectra were corrected to the excitation source intensity. The emission spectra were measured with a PMA-12 photonic multichannel analyser (Hamamatsu, Hamamatsu City, Japan) equipped with back-thinned charge-coupled device (BT-CCD) line (Hamamatsu, Hamamatsu City, Japan). As an excitation source a pulsed 266 nm line of Nd: YAG laser (3rd harmonic) and a pulsed 395 nm line from Ti: Sapphire tunable laser pumped by 532 nm line from Nd:YAG laser were used (LOTIS TII, Minsk, Belarus). The detection setup is calibrated and has flat response in whole working range (350–1100 nm). The measurements were carried out in 300 K and 80 K as well as in the temperature range 80–550 K using Linkam THMS600 cooling/heating stage (Linkam, Epsom, UK).

The luminescence kinetics were measured by using Jobin-Yvon THR1000 monochromator (HORIBA Jobin-Yvon, Palaiseu, France) equipped with Hamamatsu R928 photomultiplier (Hamamatsu, Hamamatsu City, Japan) and 1200 lines/mm grating blazed at 500 nm. As an excitation source a pulsed 395 nm line from Ti: Sapphire tunable laser (LOTIS TII, Minsk, Belarus) pumped by 532 nm line from Nd: YAG laser was used. The decay profiles were collected using a LeCroy WaveSurfer 400 MHz oscilloscope (Teledyne LeCroy, Chestnut Ridge, NY, USA). The luminescence kinetics were monitored at 612 nm line which is the maximum of the most intense electric dipole transition (^5^D_0_ → ^7^F_2_) and the effective emission lifetimes were calculated by the following equation:

(1) $$\tau_{m}\frac{\int_{0}^{\infty }\mathrm{tI}\left(t\right)\mathrm{dt}}{\int_{0}^{\infty }I\left(t\right)\mathrm{dt}}≅\frac{\int_{0}^{t^{\max}}\mathrm{tI}\left(t\right)\mathrm{dt}}{\int_{0}^{t^{\max}}I\left(t\right)\mathrm{dt}}$$

where I(t) is the luminescence intensity at time t corrected for the background, and the integrals are calculated over the range of 0 < t < t_max_, where t_max_ >> τ_m_.

A blue luminescence decay curve was excited by a femtosecond laser system Libra Series (Coherent, Wilsonville, USA; 1 mJ, 89 fs, 800 nm) coupled to a Light Conversion OPerA-Solo Optical Parametric Amplifier (Coherent, Wilsonville, USA) and a C5680 streak camera (Hamamatsu, Hamamatsu City, Japan) was used as a detector. The decay curve fitting was performed via Origin 2019 software (OriginLab, Northampton, MA, USA) by using following single exponential decay formula:

I(t) = I_0_ + A exp(−t/τ) (2)

where I(t) and I_0_ are the luminescence intensity at time t and t >> τ, A is a constant, and τ is the decay time for an exponential component.

## 3. Results and Discussion

### 3.1. Structural and Morphological Analysis

**Figure 1:**
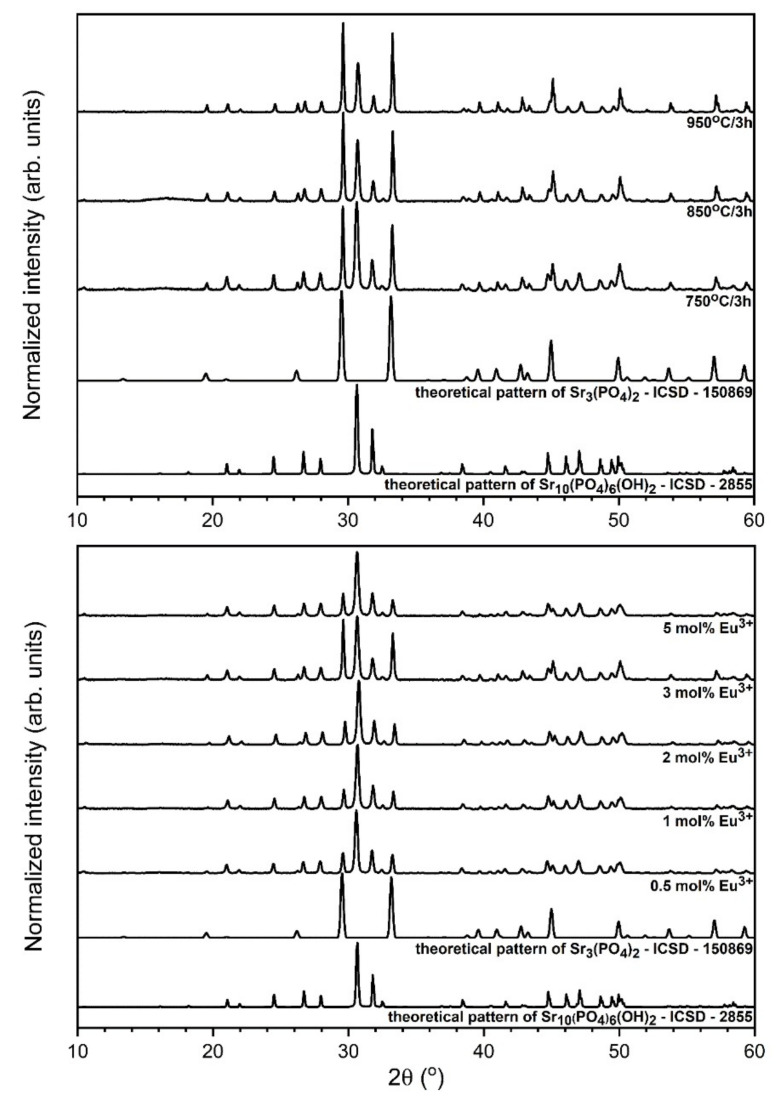
X-ray powder diffraction patterns of the Sr_10_(PO_4_)_6_(OH)_2_–Sr_3_(PO_4_)_2_ composite doped with 3 mol% Eu^3+^ ions as a function of annealing temperature (up) and annealed at 750 °C composites as a function of dopant ions concentration (bottom).

The formation of the strontium phosphate composite (Sr_10_(PO_4_)_6_(OH)_2_–Sr_3_(PO_4_)_2_) doped with x mol% Eu^3+^ ions crystalline powders (where x – 0.5, 1, 2, 3, 5 mol%) was followed by the powder XRD measurements both as a function of annealing temperature and of concentration of dopant ions (see Figure 1). The crystallinity was detected for all prepared materials sintered at different temperatures (750–950 °C per 3 h). The presence of both phases in the final product was confirmed by the correlation of the diffraction patterns with the reference standard of the hexagonal strontium hydroxyapatite ascribed to the P6_3_/m space group form Inorganic Crystal Structure Database (ICSD-2866) [27] and the trigonal tristrontium diphosphate ascribed to the R3−m space group (ICSD-150869) [28]. All the materials heat-treated at 750–950 °C have shown detectable crystallinity in the form of sharp, narrow diffraction peaks. All registered diffraction peaks were assigned to the hydroxyapatite and tristrontium diphosphate. No extra diffraction peaks were observed. The ratio of strontium phases is different and are depended on the heat-treating temperature as well as on europium(III) ions concentration (see Table 1). With an increase of annealing temperature, the content of the Sr_3_(PO_4_)_2_ crystal phase in composite increases due to the dehydration process of hydroxyapatite occurring at elevated temperature. It is not possible to detect any trend according to content of particular crystal phases when the concentration of the Eu^3+^ in composites increases. The Sr_3_(PO_4_)_2_ crystal phase amount in composite increases form 37% for 750 °C temperature to 52% for 950 °C in thermally treated materials. This phenomenon is related to dehydration process occurring in hydroxyapatite matrix under elevated temperature.

The structural refinement has been performed to get the unit cell parameters and percentage composition of the composite. The hexagonal and trigonal phases formation as well as the effective incorporation of Eu^3+^ ions have been confirmed. A good agreement between the recorded XRD pattern and the theoretical fit has been found. It indicates the success of the Rietveld refinement method. More details regarding Rietveld refinement are displayed in Table 1. As can be seen, it is possible to observe a shrinkage of both cells volume and a parameters of the SrHAp as well as the TSP materials with an increase of the Eu^3+^ ions concentration, which is caused by smaller ionic radii of dopant (Sr^2+^ (CN9)—1.31 Å, Eu^3+^ (CN9)—1.12 Å and Sr^2+^ (CN7)—1.21 Å, Eu^3+^ (CN7)—1.01 Å) as well as tristrontium diphosphate (Sr^2+^ (CN6)—1.18 Å, Eu^3+^ (CN6)—0.947 Å and Sr^2+^ (CN10)—1.36 Å, Eu^3+^ (CN9)—1.12 Å) [29]. Moreover, the a and V cells parameters of both composite materials marginally decreased after thermal treatment, which is an expected and well-known consequence caused by increase of particle size. An increase of annealing temperature caused an increase of Sr_3_(PO_4_)_2_ crystal phase content in composite.

**Figure 2:**
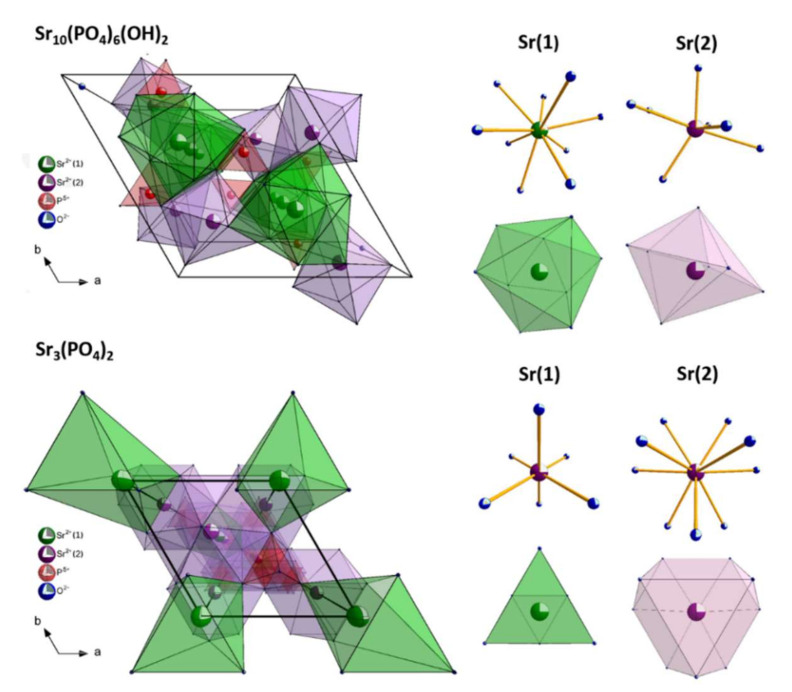
Visualization of the hexagonal strontium apatite and trigonal tristrontium diphosphate unit cells with the coordination polyhedra of the cations.

The visualization of the ideal hexagonal strontium apatite unit cell and trigonal tristrontium diphosphate unit cells as well as the coordination polyhedrals of Sr^2+^ cations is presented in Figure 2. Two different types of crystallographic sites of Sr^2+^ ions in hydroxyapatite lattice are current: four at the Sr(1) site with C_3_ point symmetry situated on the ternary axes and six at the Sr(2) site with C_S_ symmetry [30,31]. Moreover, in apatite structure, cis and trans symmetry of C_S_ crystallographic site correlated with arrangements of Eu^3+^ ions along the channel is well-known [8]. In the Sr_3_(PO_4_)_2_ matrix, there are two nonequivalent crystallographic sites of Sr^2+^ ions: three at the Sr(1) site and six at the Sr(2) site. The atoms at the Sr(1) crystallographic site are placed on the threefold axis coordinated by 6 oxygen atoms belonging to the PO_4_^3−^ group and site symmetry C_3v_. The ions at the Sr(2) site are coordinated by ten oxygen atoms having C_S_ symmetry [32]. Nevertheless, a charge and an ionic radii incompatibility between Sr^2+^ and Eu^3+^ ions are present, leading to some structural defects correlated with the charge balance necessity.

**Figure 3:**
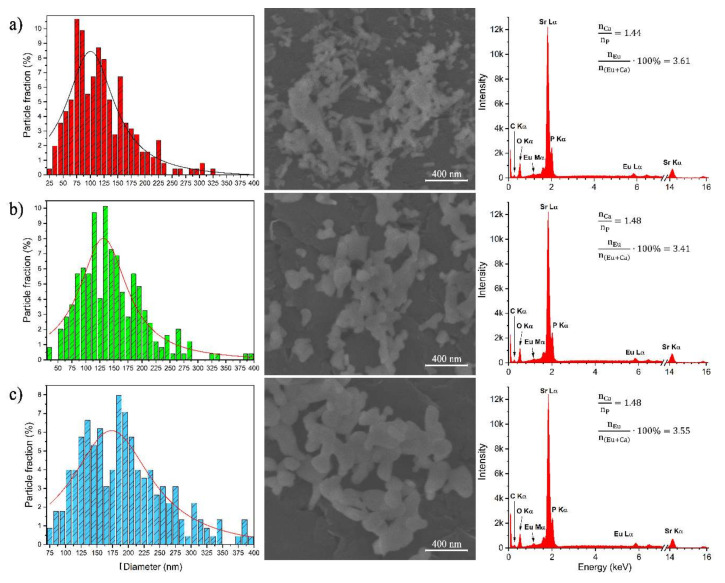
The particle size distribution (left) based on SEM images (middle) as well as EDS spectra (right) of the Sr_10_(PO_4_)_6_(OH)_2_–Sr_3_(PO_4_)_2_ composite doped with 3 mol% Eu^3+^ ions annealed at 750 (**a**), 850 (**b**) and 950 °C (**c**).

The morphology of the Sr_10_(PO_4_)_6_(OH)_2_–Sr_3_(PO_4_)_2_ composite doped with 3 mol% Eu^3+^ ions annealed at 750, 850 and 950 °C was studied using a Scanning Electron Microscope. The particles are aggregated, their shape is asymmetrical and elongated in one direction (see Figure 3). The morphology of particles was not changed with an increase of the annealing temperature. However, the particle size was changed with an increase of the annealing temperature. The particles size distributions were counted and displayed as histograms. The size distributions are relatively wide, which is caused by different grain growth speed of each phase. The average size of the Sr_10_(PO_4_)_6_(OH)_2_–Sr_3_(PO_4_)_2_ composite doped with 3 mol% Eu^3+^ annealed at 750, 850 and 950 °C are about 100 (± 3.4), 131 (± 2.6) and 173 nm(± 4.6 nm), respectively. The energy-dispersive X-ray spectroscopy was used to confirm the content of elements in obtained materials. 

**Figure 4:**
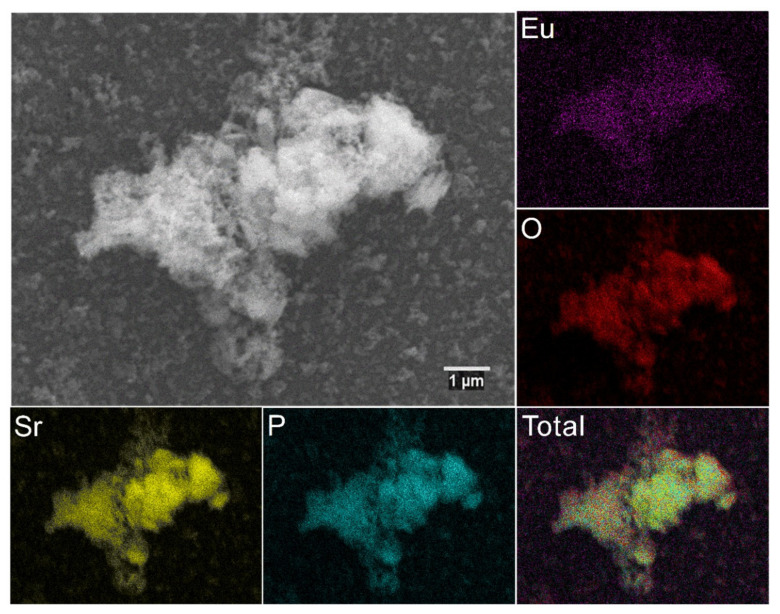
SEM image and EDS elemental maps of the 3 mol% Eu^3+^:Sr_10_(PO_4_)_6_(OH)_2_–Sr_3_(PO_4_)_2_ composite annealed at 750 °C.

The element maps were recorded by the SEM-EDS technique to analyze the elements distribution in the obtained material (Figure 4). The results confirmed the stated stoichiometry of dopants and regular distribution of elements.

**Figure 5:**
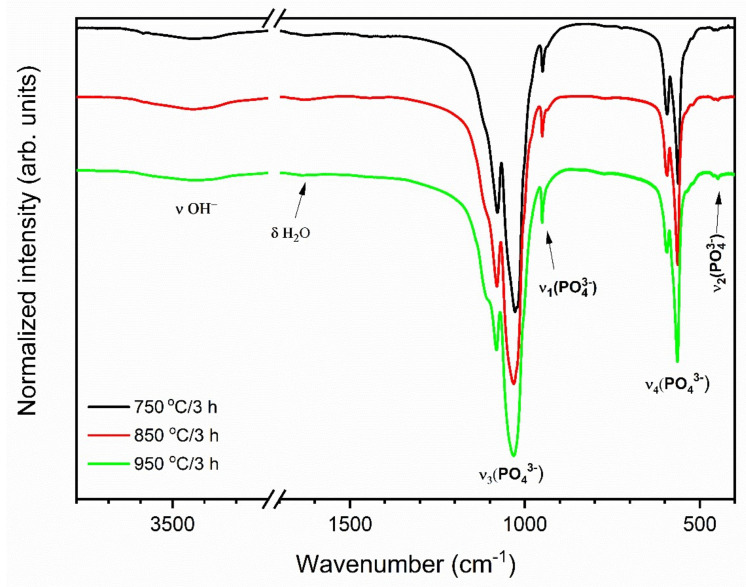
FT-IR spectra of the annealed 3 mol% Eu^3+^: Sr_10_(PO_4_)_6_(OH)_2_–Sr_3_(PO_4_)_2_ composite annealed at different temperatures.

The FT-IR spectra were measured to identify the existence of orthophosphate and hydroxyl groups in obtained materials (Figure 5). The infrared spectra consist of ordinary PO_4_^3-^ vibration bands: the v_2_ bending at 448 cm^−1^; 564 cm^−1^, the v_4_ vibration at 596 cm^−1^; the v_1_ symmetric stretching at 950 cm^−1^; the v_3_ antisymmetric stretching at 1033 cm^−1^ and 1081 cm^−1^. Moreover, the vibrational transitions observed at 1632 cm^−1^ 3432 cm^−1^ (v OH) confirm the presence of a hydroxyl group in the obtained materials [33,34,35].

### 3.2. Emission and Excitation Spectra

**Figure 6:**
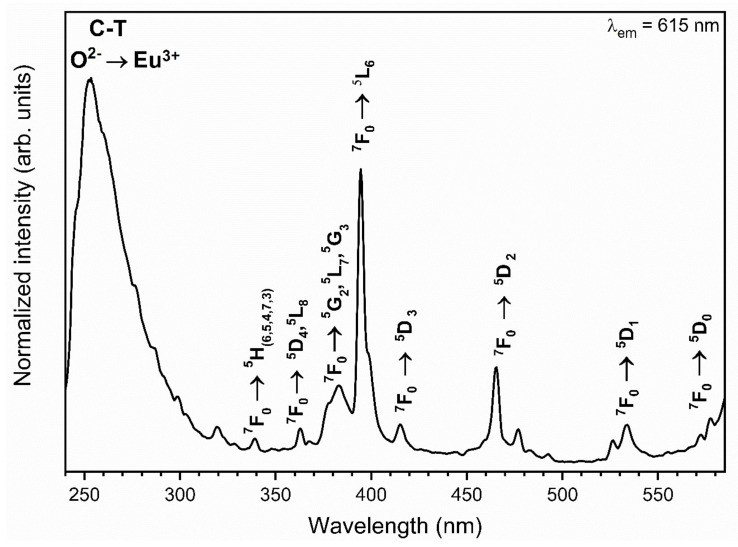
Representative excitation-emission spectra of the Sr_10_(PO_4_)_6_(OH)_2_–Sr_3_(PO_4_)_2_ composite doped with 1 mol% Eu^3+^ ions annealed at 750 °C.

The synthesised materials’ excitation-emission spectra were measured at room temperature and observed at 615 nm emission wavelength of the maximum of the most intense ^5^D_0_ → ^7^F_2_ electric dipole transition. All recorded spectra were alike to each other, thus only the representative excitation spectra of 1 mol% Eu^3+^: Sr_10_(PO_4_)_6_(OH)_2_–Sr_3_(PO_4_)_2_ annealed at 750 °C was presented in Figure 6. The weak intraconfigurational 4f-4f transitions with sharp lines are characteristic for the Eu^3+^ ions as well as the intense ligand-to-metal charge transfer (CT) O^2−^ → Eu^3+^ with broad band in the UV range are typical for the Eu^3+^ ions incorporated into oxide matrix. The narrow lines observed at 339.5 nm (29,445 cm^−1^) were attributed to the ^7^F_0_ → ^5^H_(6,5,4,7,3)_ transitions, at 363 nm (27,548 cm^−1^) to the ^7^F_0_ → ^5^D_4_, ^5^L_8_, the maximum at 383.5 nm (26,076 cm^−1^) to the ^5^L_8_, ^7^F_0_ → G_2_, ^5^L_7_, ^5^G_3_, at 394.3 nm (25,361 cm^−1^) to the ^7^F_0_ → ^5^L_6_, at 415.8 nm (24,050 cm^−1^) to the ^7^F_0_ → ^5^D_3_, at 465.3 nm (21,492 cm^−1^) to the ^7^F_0_ → ^5^D_2_, at 533.3 nm (18,751 cm^−1^) to the ^7^F_0_ → ^5^D_1_ and at 572 nm (17,483 cm^−1^) to the ^7^F_0_ → ^5^D_0_ transition. It is well known that the 4f-sub-shells of lanthanide ions (RE^3+^) are well protected by the 5s and 5p sub-shells and the energy levels of the 4f-electrons are weakly influenced by the RE^3+^ ion vicinity. These transitions are forbidden by Laporte parity rule that is why the f-f transition intensities are weak. However, quite the opposite, the charge-transfer (CT) transition is allowed and strongly affected by electron-lattice coupling. Moreover, its energy level is depended on an ion local symmetry [36,37,38]. The CT maximum is located at 253 nm (39,526 cm^−1^).

**Figure 7:**
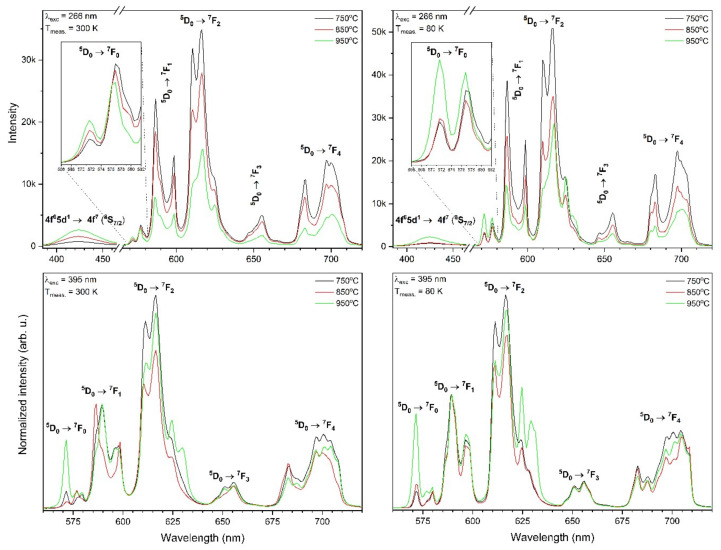
Emission spectra of the Sr_10_(PO_4_)_6_(OH)_2_–Sr_3_(PO_4_)_2_ composite doped with 3 mol% Eu^3+^ ions as a function of post-heat treatment temperature excited by 266 nm (above) and by 395 nm line (below) measured at 300 K (left) and 80 K (right).

**Figure 8:**
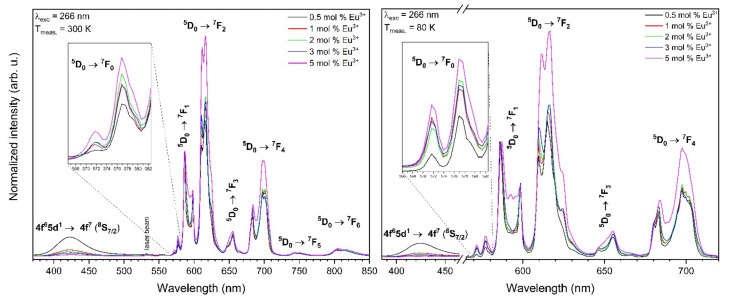
Emission spectra of the Sr_10_(PO_4_)_6_(OH)_2_–Sr_3_(PO_4_)_2_ composite doped with Eu^3+^ ions as a function of the Eu^3+^ ions concentration annealed at 750 °C, excited by 266 nm line, measured at 300 K (left) and 80 K (right).

The emission spectra of xEu^3+^: Sr_10_(PO_4_)_6_(OH)_2_–Sr_3_(PO_4_)_2_ (where x – 0.5–5 mol%) materials annealed at the temperature 750, 850 and 950 °C were recorded upon pulsed 266 nm and 395 nm excitation at 300 K as well as at 80 K (Figure 7) and as a function of Eu^3+^ ions concentration excited by 266 nm at 300 K and 80 K (Figure 8). The spectra excited by 395 nm line were normalized to the ^5^D_0_ → ^7^F_1_ transition, which can be treated as an internal reference. In the emission spectra measured upon excitation by 266 nm, a broad peak at about 421 nm was observed, which corresponds to the 4f^6^5d^1^ → 4f^7^ (^8^S_7/2_) emission of Eu^2+^ ions as well as narrow 4f-4f transitions were present, which are typical for Eu^3+^ ions. As can be seen, an increase of the annealing temperature results in an increase of the Eu^2+^ ions emission intensity and a decrease of Eu^3+^ emission intensity (see Figure 7 upside). The Eu^2+^ emission measured at 80 K is less intense compared to the Eu^2+^ emission measured at 300 K. A change in the annealing temperature and measurement temperature results in changes of the Eu^2+^ to Eu^3+^ relative intensity ratio. Moreover, the intensity of the Eu^2+^ ions emission depends on the measurement temperature and it is more intense in higher temperatures (see Figure 7). This was caused by an increase of Eu^2+^ fraction in the composite. The detailed description of reduction mechanism is provided in Section 3.3 and Section 3.4.

The Eu^3+^ emission spectra consist of characteristic electron transitions occurring in the 4f shells appearing in the red region of the electromagnetic radiation. The ^5^D_0_ →^7^F_0_ transition is located at 576.9 nm (17,334 cm^−1^), the ^5^D_0_ → ^7^F_1_ transition at 585.9 nm (17,068 cm^−1^), the ^5^D_0_ → ^7^F_2_ transition at 616.5 nm (16,221 cm^−1^), the ^5^D_0_ → ^7^F_3_ transition at 655.2 nm (15,262 cm^−1^), the ^5^D_0_ → ^7^F_4_ transition at 700.5 nm (14,275 cm^−1^), the ^5^D_0_ → ^7^F_5_ transition at 742.7 nm (13,464 cm^−1^) and the ^5^D_0_ → ^7^F_6_ transition at 805.3 nm (12,418 cm^−1^). The ^5^D_0_ → ^7^F_0_ transition is split into three components located at 571.6 nm (17,495 cm^−1^), 576.9 nm (17,334 cm^−1^) and 579.9 nm (17,244 cm^−1^), indicating that Eu^3+^ ions are located at least into three different crystallographic sites in this composite [39,40,41,42]. A difference between the intensity ratio of components is visible. 

The hypersensitive ^5^D_0_ → ^7^F_2_ emission transition is the most intense in all cases. The intensity of this transition is very sensitive to variations in the local environment of Eu^3+^ ions in the host. On the other hand, the intensity of the ^5^D_0_ → ^7^F_1_ magnetic dipole transition remains almost independent on the crystal field. When Eu^3+^ ions occupy a centrosymmetric site, the only permissive transition is magnetic one. In the opposite, the electric dipole transition is cardinal. The asymmetry ratio of the relevant emission intensities (R) is defined followingly [43]:

(3) $$R=\;\frac{\int \;{}^{5}D{}_{0}\to {}^{7}F{}_{2}}{\int \;{}^{5}D{}_{0}\to {}^{7}F{}_{1}}$$

The integrated intensities ratio of these transitions is used to evaluate the asymmetry of the Eu^3+^ ions coordination polyhedron. The higher the relationship between these transitions is the less centrosymmetric the local environment of Eu^3+^ ions becomes. The annealing temperatures and Eu^3+^ ions concentration impacts on the R value is gathered in Table 2. Any relationship between an increase of annealing temperature and dopant concentration is not observed. The crystallography sites in phosphate lattice are randomly occupied by Eu^3+^ ions what is related to the Eu^3+^ ion concentration and annealing temperature. Moreover, the observed emission is related to different crystallographic sites. That is why the R value behaves incalculably in such cases.

The Stark’s components are better visible in the case of emission spectra measured at 80 K (see Figure 7). The emission spectra upon excitation by 395 nm line were also measured to be compared. As can be seen, some differences between these two-excitation lines are well visible as different shapes, ratios, and intensity of transitions. The 3 mol% Eu^3+^: Sr_10_(PO_4_)_6_(OH)_2_–Sr_3_(PO_4_)_2_ material stands out by the abnormal high intensity of the ^5^D_0_ → ^7^F_0_ transition upon excitation by the 395 nm line.

The emission spectra of the Eu^3+^-doped Sr_10_(PO_4_)_6_(OH)_2_–Sr_3_(PO_4_)_2_ composite depend on the Eu^3+^ ions concentration measured upon 266 nm excitation at 300 and 80 K is presented in the Figure 8 As can be observed, an increase of the europium ions concentration in material led in a decrease of the photoluminescence (PL) intensity of Eu^2+^ ions. This insight can be caused by the Eu^2+^ → Eu^3+^ energy transfer, which prompts in the quenching of the Eu^2+^ emission. In materials doped with low Eu^3+^ ions concentration, the Eu^2+^ ions are weakly quenched due to the statistically enormous distance between these two ions. An increase of dopant concentration caused an increase of the Eu^2+^ → Eu^3+^ energy transfer efficiency as a result of a bigger concentration of ions in the host [7,44]. This could also be caused by the different emission intensity in two compounds due to the change of Sr_10_(PO_4_)_6_(OH)_2_ to Sr_3_(PO_4_)_2_ proportion with an increase of Eu ions doping.

On the other hand, the decrease of Eu^2+^ PL intensity may be clarified by the fact that the amount of V″_Sr_ vacancies also increase with an increase of the Eu^3+^ concentration and an excess of the Eu^3+^ ions would occupy the V″_Sr_ position. Consequently, the double negative vacancy (V″_Sr_) would be converted into a positive charge hereupon, it cannot move negative charge to Eu^3+^ and the reduction was inhibited. To have electroneutrality, the composite may absorb oxygen from air.

The Ω_2_ and Ω_4_ intensity parameters was calculated based on the Judd–Ofelt theory and the results are listed in Table 2. The Ω_2_ parameter value changes lightly with an increase of Eu^3+^ ion concentration and with an increase of annealing temperature, but without any well visible tendency. It indicates the changes in the europium coordination polyhedra and could be related to changes of the Eu^3+^–O^2−^ bond covalency. The Ω_4_ value brings information about the electron density variations around Eu^3+^ cations. However, the Eu^3+^ ions symmetry changes cannot be directly interpreted by the Ω_4_ value but can add some information about the electron density deviations in the O^2−^ anions surrounding, what influences on the CT band position.

### 3.3. Abnormal Reduction Mechanism of Eu^3+^ → Eu^2+^

The abnormal reduction of the Eu^3+^ to the Eu^2+^ ion is known in the literature for solid-state materials made at elevated temperature in air [45,46,47]. Su et al. introduced four demands for these abnormal reductions of the Eu^3+^ ions to the Eu^2+^ ions in a solid-state host:

(1) no oxidizing ions are present in compounds,
(2) the Eu^3+^ ions substitute the cations with lower valences in compounds,
(3) similar radii of the substituted cation and Eu^2+^ ion, and
(4) the tetrahedral anion groups (BO_4_, PO_4_, AlO_4_, SiO_4_) are present in compound [47].

In the Sr_10_(PO_4_)_6_(OH)_2_–Sr_3_(PO_4_)_2_ composite, the Sr^2+^ and P^5+^ are not oxidizing ions, which satisfied condition (1). The Eu^3+^ ions substituted Sr^2+^ sites in this composite, which means that Eu^3+^ ions substituted cations with lower valences. Moreover, the Sr^2+^ ion has similar radii to Eu^2+^ (in hydroxyapatite: Sr^2+^ (CN9)—1.31 Å, Eu^2+^ (CN9)—1.30 Å and Sr^2+^ (CN7)—1.21 Å, Eu^2+^ (CN7)—1.20 Å), in tristrontium diphosphate: Sr^2+^ (CN6)—1.18 Å, Eu^2+^ (CN6)—1.17 Å and Sr^2+^ (CN10)—1.36 Å, Eu^3+^ (CN9)—1.35 Å [29]) and meet conditions (2) and (3). Furthermore, the condition (4) is also achieved, because this composite is built by PO_4_^3-^ anions groups. Based on this conditions and emission spectra, it is expected that the Eu^3+^ to Eu^2+^ reduction occur in these composites. The Eu^3+^ ions were partially reduced to the Eu^2+^ and this reduction in composites obtained in air atmosphere at elevated temperature can be clarified by the charge compensation mechanism based on the Kröger–Vink-notation (^*^ sign means charge neutrality, ^•^ means positive charge and ^′^ negative charge in particular crystallographic site. The vacancy is marked by V and interstitial atom by _i_). When the trivalent Eu^3+^ ions substituted the Sr^2+^ ions, two Eu^3+^ ions would substitute for three Sr^2+^ ions to preserve charge electroneutrality and a double negative vacancy in the Sr crystallographic site (V″_Sr_) is created. Consequently, two Eu^•^_Sr_ positive defects and one V″_Sr_ negative vacancy would be designed by each substitution for every two Eu^3+^ ions:

Eu_2_O_3_ + 3Sr^*^_Sr_ → 2(Eu^3+^)^•^_Sr_ + V″_Sr_ + 3CaO; (4)

Two relative negative charges are composed on the hydroxyl sides:

Eu_2_O_3_ + 2Ca^*^_Ca_ + 2OH^*^_OH_ → 2(Eu^3+^)^•^_Ca_ + 2O^′^_OH_ + 2CaO + H_2_O; (5)

In addition, charge balance can also be reached by the creation of interstitial oxygen O″_i_ with double relative negative charge:

Eu_2_O_3_ + 2Ca^*^_Ca_ → 2(Eu^3+^)^•^_Ca_ + O″_i_ + 2CaO; (6)

The vacancy V″_Sr_ could behave as a donor of electrons pending the two Eu^•^_Sr_ defects turn into acceptors of electrons. Via thermal stimulation, the negative charges of the V″_Sr_ vacancy defects would be thereafter moved to the Eu^3+^ positions and reduce them to their Eu^2+^ form followingly:

2(Eu^3+^)^•^_Sr_ + V″_Sr_ → 2(Eu^2+^)^*^_Sr_ + V^*^_Sr_; (7)

The higher annealing temperature, the mechanism of the Eu^3+^ to Eu^2+^ reduction is more probable, which is well visible on the emission spectra.

### 3.4. Thermal Stability

**Figure 9:**
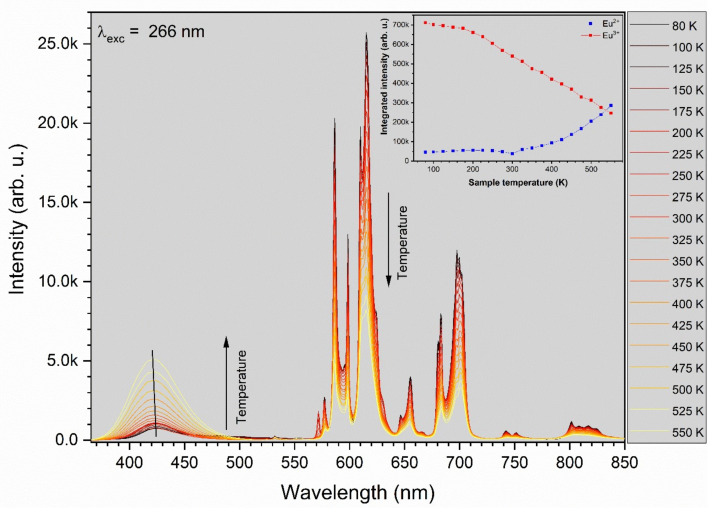
The luminescence intensity dependence on the measurement temperature of 1 mol% Eu^3+^: Sr_10_(PO_4_)_6_(OH)_2_–Sr_3_(PO_4_)_2_ in the temperature range of 80–550 K. Inset: dependence of integrated intensity of the Eu^2+^ and Eu^3+^ emission on the measurement temperature.

**Figure 10:**
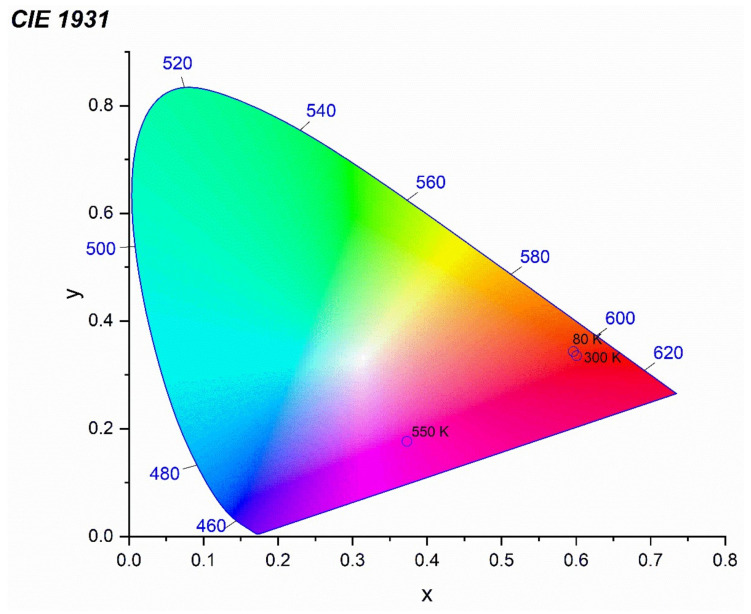
CIE chromaticity coordinates values of the 1 mol% Eu^3+^: Sr_10_(PO_4_)_6_(OH)_2_–Sr_3_(PO_4_)_2_ excited by 266 nm and measured in 80, 300 and 550 K of temperature.

The luminescence intensity of the 1 mol% Eu^3+^: Sr_10_(PO_4_)_6_(OH)_2_–Sr_3_(PO_4_)_2_ was measured depending on the measurement temperature in the range of 80–550 K (see Figure 9). It was observed that the PL intensity of Eu^3+^ ion decreases with an increase of the measurement temperature but on the other hand the PL intensity of Eu^2+^ ion increases. Mechanism of this behavior has been described as abnormal intense Eu^2+^ emission in high temperature [16,48]. It could be related to the presence of two possible defect traps of [2(Eu^3+^)^•^_Sr_ − O_i_″] or [2(Eu^3+^)_Sr_^•^ − V_Sr_″] in this material. The Eu^3+^ ions could recombined with the electrons released from defects O_i_″ or V_Sr_″; nevertheless, the electrons do not get into the Eu^2+^ ion but loosely couple with Eu^3+^ in the near environment (Eu^3+^ + e^−^) manifesting emission of Eu^2+^. All the defects can be inert at low temperature what yield low luminescence of Eu^2+^ ion when the temperature decrease. The CIE 1931 chromaticity diagram is presented in the Figure 10 determined from emission spectra measured in 80, 300 and 550 K of temperature.

### 3.5. Fluorescence Dynamics

**Figure 11:**
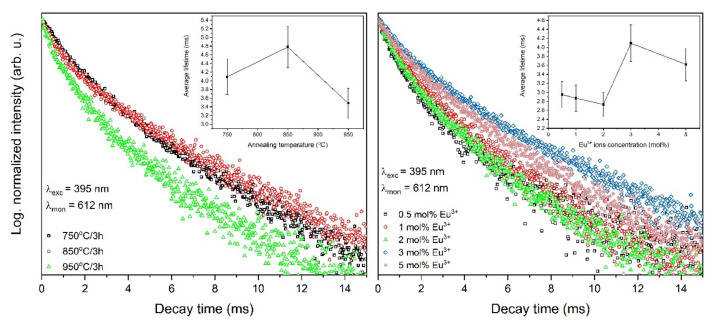
Luminescence kinetics of the Sr_10_(PO_4_)_6_(OH)_2_–Sr_3_(PO_4_)_2_ composite doped with 3 mol% Eu^3+^ ions as a function of heat-treatment temperature (left) and annealed composite at 750 °C as a function of europium ions concentration (right).

The luminescence kinetics were analyzed for all synthesized materials to determine the comprehensive characteristics of the luminescence properties. The decay times at room temperature (RT) corresponding to the ^5^D_0_ → ^7^F_2_ transition were excited by pulse radiation of 395 nm and monitored at 615 nm (Figure 11). The decay curves are not single-exponential, what is compatible with the presence of nonequivalent crystallographic sites of Eu^3+^ ions accordingly, the lifetimes values were calculated as the effective emission decay time by using Equation (1). The decay profiles and the lifetime values are alike to each other and only minor differences were observed. The decay times of Eu^3+^ ions in Sr_10_(PO_4_)_6_(OH)_2_–Sr_3_(PO_4_)_2_ are typical for this kind of ion and are longer than for strontium apatite [7,8], which is caused by the presence of Sr_3_(PO_4_)_2_ phase.

**Figure 12:**
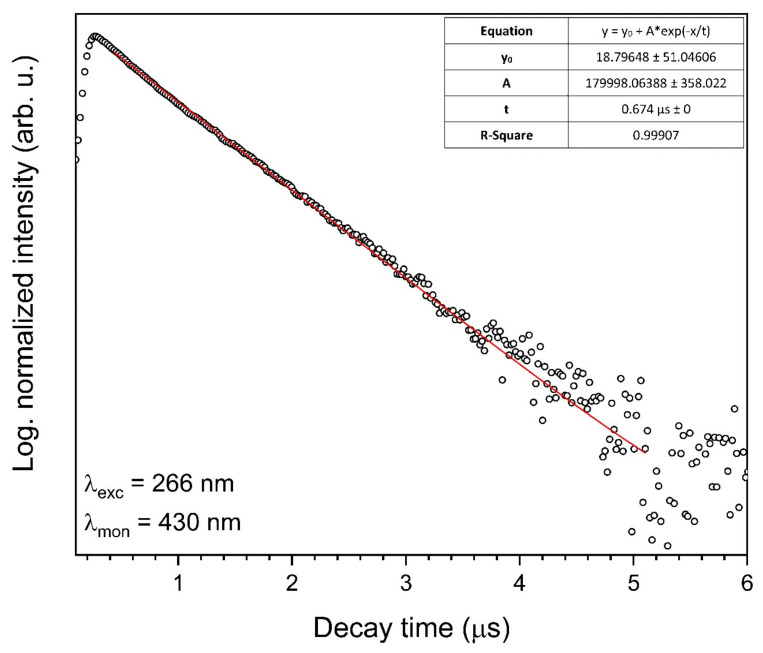
Luminescence kinetics of the blue component of the 1 mol% Eu^3+^: Sr_10_(PO_4_)_6_(OH)_2_–Sr_3_(PO_4_)_2_ composite annealed at 750 °C. Excited by 266 nm and observed at 430 nm.

**Figure 13:**
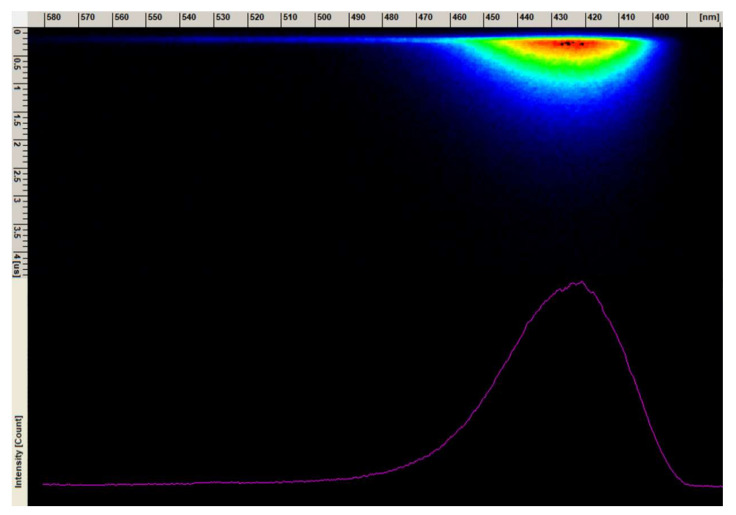
The emission spectrum image form streak camera of the 1 mol% Eu^3+^: Sr_10_(PO_4_)_6_(OH)_2_–Sr_3_(PO_4_)_2_ composite annealed at 750 °C.

The decay curve (blue component) of the 1 mol% Eu^3+^: Sr_10_(PO_4_)_6_(OH)_2_–Sr_3_(PO_4_)_2_ composite annealed at 750 °C was measured under excitation at 266 nm and monitored at 430 nm (Figure 12). The single exponential shape of the decay curve indicates to the Eu^2+^ ion emission from one crystallographic site. The lifetime value was calculated based on Equation (2) and was determined to be 0.674 µs. The emission spectra of blue emission from the streak camera is presented in Figure 13.

## 4. Conclusions

A microwave-assisted hydrothermal method was used with success to produce Eu^3+^-doped Sr_10_(PO_4_)_6_(OH)_2_–Sr_3_(PO_4_)_2_ composites. The structural and luminescence properties were investigated in a function of dopant ions concentration and annealing temperature. The formation of the strontium phosphate composite was confirmed by the XRPD analysis. The unit cell parameters as well as the percentage content of crystal phases, were calculated by Rietveld refinement method. The obtained materials have had a tendency to agglomerate to minimize their excess surface energy and their shapes were irregular and elongated. There were no observed changes in morphology with an increase of the annealing temperature. There were observed the narrow 4f-4f transitions typical for Eu^3+^ ions and a broadband at 421 nm related to the 4f^6^5d^1^→ 4f^7^(^8^S_7/2_) transition from Eu^2+^ ions. Moreover, it has been described as an abnormal intense emission of Eu^2+^ ion due to recombination of the excited state of Eu^3+^ ion with the electrons released from defects (O_i_″ or V_Sr_″).

The decay curves of Eu^3+^ ions are not single-exponential as a result of the nonequivalent crystallographic sites of Eu^3+^ ions in the matrix. However, the lifetime value of Eu^2+^ ions is single-exponential and was determined to be 0.674 µs.

## Figures and Tables

**Table 1:** Unit cell parameters (a and c), crystal cell volume (V), the percentage of particular phases calculated based on the Rietveld methods, and refined factors (R_w_) presented for the Sr_10_(PO_4_)_6_(OH)_2_–Sr_3_(PO_4_)_2_ powder composites as a function of dopant concentration and sintering temperature.

Sample	Cell Parameters						Phase		
	Belovite Sr_5_(PO_4_)_3_OH			Tristrontium Bis(Phosphate(V)) Sr_3_(PO_4_)_2_			SrHAp (%)	TSP (%)	R_w_
	a (Å)	c (Å)	V (Å^3^)	a (Å)	c (Å)	V (Å^3^)			
**s. c.**	9.745 (1)	7.265 (1)	597.49 (15)	5.3975 (2)	19.818 (3)	500.01 (8)	–	–	–
**0.5% mol Eu^3+^; 750** **°C**	9.769 (1)	7.282 (1)	601.84 (07)	5.3912 (5)	19.794 (9)	498.23 (6)	78.16	21.84	1.38
**1% mol Eu^3+^; 750** **°C**	9.767 (3)	7.282 (1)	601.59 (43)	5.3913 (1)	19.798 (1)	498.35 (5)	82.79	17.21	1.30
**2% mol Eu^3+^; 750** **°C**	9.769 (2)	7.278 (5)	601.51 (01)	5.3921 (2)	19.802 (6)	498.60 (4)	80.36	19.64	1.20
**3% mol Eu^3+^; 750** **°C**	9.758 (9)	7.270 (1)	599.49 (65)	5.3892 (6)	19.795 (8)	497.89 (1)	62.65	37.35	1.28
**5% mol Eu^3+^; 750** **°C**	9.760 (5)	7.270 (1)	599.74 (22)	5.3886 (5)	19.796 (7)	497.80 (6)	80.55	19.45	1.55
**3% mol Eu^3+^; 850** **°C**	9.748 (5)	7.262 (7)	597.61 (01)	5.3904 (1)	19.798 (7)	498.18 (9)	50.81	49.19	2.12
**3% mol Eu^3+^; 950** **°C**	9.735 (1)	7.251 (1)	595.11 (44)	5.3904 (5)	19.796 (4)	498.13 (8)	47.69	52.31	1.46

**Table 2:** Decay rates of radiative (A_rad_), nonradiative (A_nrad_) and total (A_tot_) processes of the ^5^D_0_ → ^7^F_J_ transitions, luminescence lifetimes (T), intensity parameters (Ω_2_, Ω_4_), quantum efficiency (η) and asymmetry ratio (R) of the Sr_10_(PO_4_)_6_(OH)_2_–Sr_3_(PO_4_)_2_ composites.

3 mol% Eu^3+^: Sr_10_(PO_4_)_6_(OH)_2_–Sr_3_(PO_4_)_2_								
Sample	A_rad_ (s^−1^)	A_nrad_ (s^−1^)	A_tot_ (s^−1^)	T (ms)	Ω_2_ (10^20^cm^2^)	Ω_4_ (10^20^cm^2^)	η (%)	R
750 °C	167.56	86.11	253.67	3.94	3.2704	3.2757	66.05	2.321
850 °C	154.13	73.81	227.94	4.39	2.9401	2.9301	67.62	2.087
950 °C	164.66	150.97	315.63	3.17	3.3232	2.9414	52.17	2.359
**x mol% Eu^3+^:Sr_10_(PO_4_)_6_(OH)_2_–Sr_3_(PO_4_)_2_ 750 °C**								
0.5 mol% Eu^3+^	182.37	259.13	441.50	2.27	3.4835	3.9733	41.31	2.473
1 mol% Eu^3+^	199.03	190.38	389.41	2.57	4.0504	4.0731	51.11	2.875
2 mol% Eu^3+^	187.74	218.58	406.32	2.46	3.7431	3.8447	46.21	2.657
3 mol% Eu^3^	167.56	86.11	253.67	3.94	3.2704	3.2757	66.05	2.321
5 mol% Eu^3+^	185.04	130.41	315.45	3.17	3.7257	3.6728	58.66	2.645
